# Hybrid aortic arch replacement with a modified stent bridging for the left subclavian artery

**DOI:** 10.1016/j.xjtc.2026.102307

**Published:** 2026-03-07

**Authors:** Federico Giorgi, Laura Besola, Andrea Colli

**Affiliations:** Cardiac Surgery Division, Department of Surgical, Medical and Molecular Pathology and Critical Care, University of Pisa, Pisa, Italy

**Figure:**
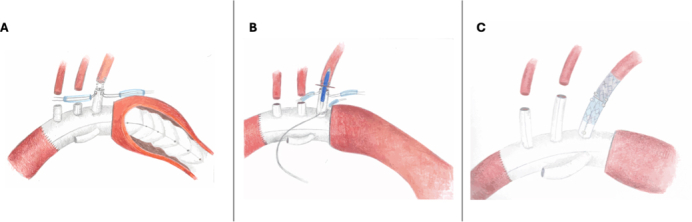
Modified Supra Aortic Vessel anastomosis STEnt Bridging (mSAVSTEB) technique.

**Central Message**

A modified SAVSTEB technique enables faster, sutureless LSA management during FET procedures, reducing ischemia time while maintaining reliable perfusion and hemostasis.

Aortic arch replacement for dissection and aneurysm has advanced with the frozen elephant trunk (FET) technique and antegrade cerebral perfusion.^1^, ^2^, ^3^, ^4^ Managing supra-aortic vessels, particularly the left subclavian artery (LSA), remains challenging because of the need to preserve distal perfusion, ensure hemostasis, and prevent neurologic injury. Alternatives to direct LSA reimplantation include extra-anatomical bypass, stent bridging, direct thoracic stent fenestration with LSA stenting,^5^ and premade branched prostheses.^6^ We describe a modification of the Supra-Aortic Vessel anastomosis STEnt Bridging (SAVSTEB) technique,^E1^ aimed at reducing ischemia time and improving reproducibility. Institutional review board approval was waived. The patient provided written informed consent for the publication of study data.

## Case Presentation

**Figure 1:**
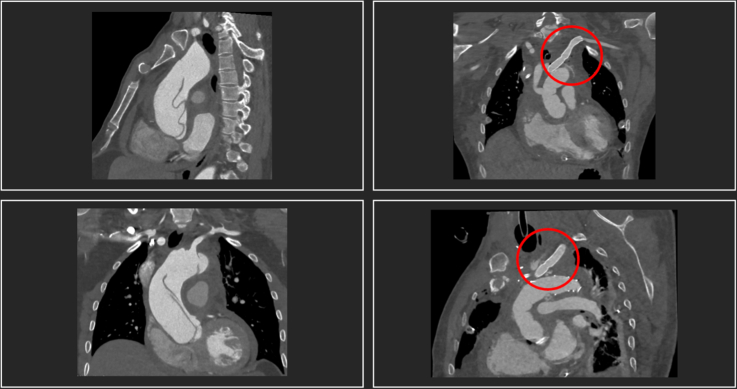
Preoperative (*left top* and *left bottom*) and postoperative (*right top* and *right bottom*) angio-computed tomography scans. *Red circle* demonstrates the VIABAHN in the left subclavian artery.

A 59-year-old man presented with acute Stanford type A aortic dissection extending from the aortic root to the femoral arteries bilaterally (Figure 1). Emergency surgery was performed via full sternotomy. Cardiopulmonary bypass was established using femoral and atriocaval cannulation. The right subclavian artery was perfused using an OptiFlow 14-Fr cannula, and the left common carotid artery with a Biomedicus 12-Fr cannula.

After aortic crossclamping, cardiac arrest was achieved with cold blood St. Thomas II cardioplegia. A Bentall procedure was performed using a 25-mm On-X mechanical root prosthesis (On-X Life Technologies). At 32 °C, circulatory arrest was initiated with antegrade cerebral perfusion. The distal ascending aorta was opened, and the brachiocephalic trunk, left common carotid artery, and LSA ostia were isolated. A balloon-tipped RDB Cannula True Flow, (MED-EUROPE) was advanced into the LSA to complete antegrade cerebral perfusion.

**Figure 2:**
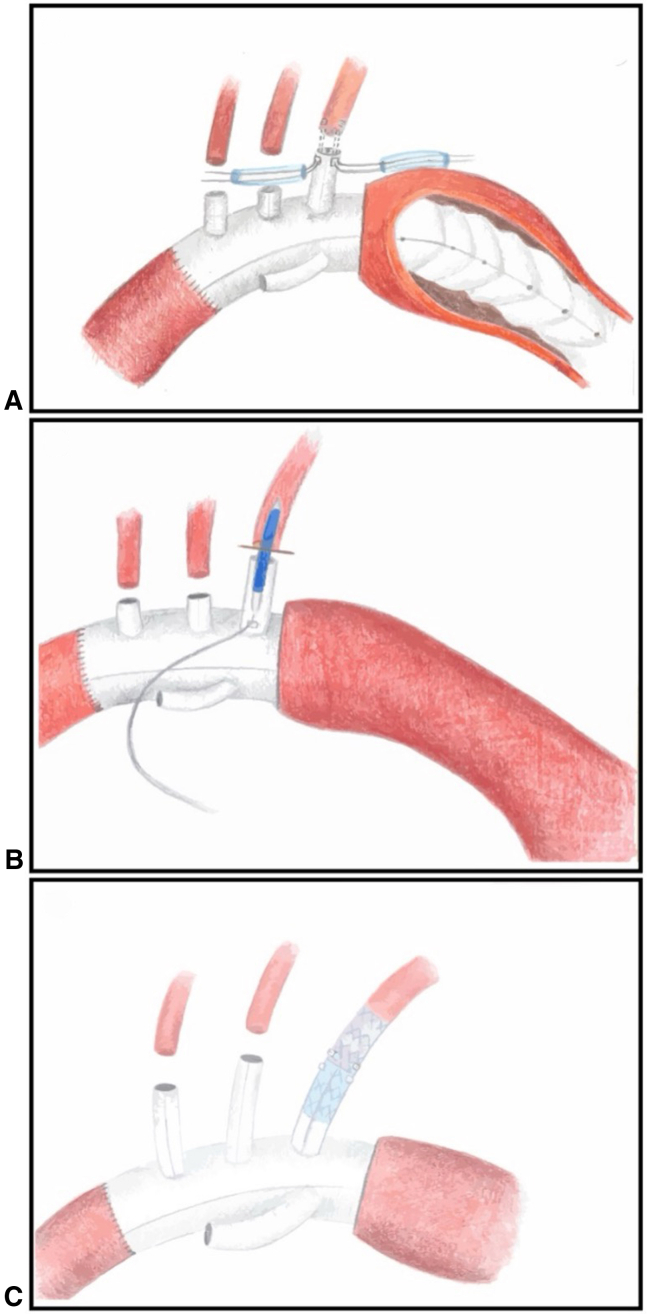
Self-expandable stent deployment in LSA vignette. A, 8-mm graft of the Thoraflex prosthesis was prepared with a U-stitch purse-string just a few millimeters distal to the proximal clamp. B, The graft branch was approximated and secured to the LSA with 2 stabilizing U-stitches with rubber tourniquets. A small cut was performed using a no. 11 blade, and a 9 × 75-mm VIABAHN self-expandable stent was inserted and advanced into the LSA up to its midportion, premarked to avoid vertebral artery compromise. C, After securing the purse-string and tying down the 2 U-stitches, LSA perfusion was restored without bleeding. *LSA*, Left subclavian artery.

A 28 × 26 × 150-mm Thoraflex arch prosthesis (Terumo Aortic) was deployed in zone 3, and distal anastomosis was completed using a Teflon strip. Systemic perfusion was restored via the “ante-flow branch,” followed by proximal prosthesis-to-prosthesis anastomosis, aortic clamp removal, and heart reperfusion. The 8-mm Thoraflex branch was prepared with a U-stitch purse-string a few millimeters distal to the proximal clamp and, after removal of RDB cannula, was secured to the LSA with 2 stabilizing U-stitches with rubber tourniquets. A small cut was performed using an 11-blade, and a 9 × 75-mm VIABAHN self-expandable stent (W. L. Gore & Associates) was inserted and advanced into mid-LSA, premarked to avoid vertebral artery compromise (Figure 2). After securing the purse-string and tying down the U-stitches, LSA perfusion was restored without bleeding. LCC and brachiocephalic trunk anastomoses were completed. Postoperative recovery was uneventful, and CT confirmed full stent deployment (Figure 1).

## Discussion

Supra-aortic vessel management ranges from conventional sutured anastomoses to extra-anatomic bypasses and stent-based sutureless anastomoses. The original Supra-SAVSTEB technique involves LSA stent implantation during circulatory arrest via a J-shaped guidewire, with slight stent protrusion into the arch graft.^E1^ Our modification reduces systemic circulatory time, simplifies assessment of graft length, and eliminates the need for a guidewire. A similar approach has been proposed for end-to-end sutureless anastomosis (VIABAHN-assisted sutureless anastomosis) in complex vascular repairs.^E2^

Main advantages include the following:

1. Performing stent bridging after restoring systemic perfusion reduces ischemia, allowing warmer temperatures (>30 °C) without compromising protection.^E3^
2. Branch length sizing is easier once the main body is pressurized after crossclamp removal.
3. Midstent marking with a sterile blue pen improves measurement reliability and vertebral artery takeoff protection.
4. Avoiding the use of guidewires reduces distal injury risk and removes the need for fluoroscopy. Back-bleeding during stent deployment is minimal and manageable, reducing the risk of air embolization once antegrade flow is reestablished.
5. Precise stent deployment and reducing the risk of migration or bleeding are warranted by the use of the 2 stabilizing U-stitches with rubber tourniquets that secure the graft branch to the LSA. Sutureless anastomosis with VIABAHN stents is highly hemostatic and prevents compression or kinking of the surgical branch.

An alternative to avoid creating a small graft incision would be to use the arch-graft side-branch for stent delivery. However, in our practice, this branch is connected to CPB immediately after completing the distal anastomosis to restore antegrade perfusion as early as possible; using it for VIABAHN deployment could therefore prolong ischemia time. If an additional branch were available, such as one dedicated to supra-aortic vessels in the setting of a bovine brachiocephalic trunk, or by adding a Y-branch to an existing limb, deployment through such a dedicated branch could streamline the procedure and enable even earlier supra-aortic reperfusion.

During modified SAVSTEB, stent sizing is critical: oversizing should not exceed 1.5 mm to balance hemostasis and vessel safety. In terms of length, we have learned that 20 mm of telescopic overlap between the stent and the native vessel, as well as the surgical branch, is considered adequate. Given the average distance of the vertebral artery takeoff from the LSA origin, which is approximately 40 mm, a 25- to 30-mm stent within the native vessel should be sufficient in most cases. This means the stent could range in length from 50 to 75 mm.

Although our experience is limited to VIABAHN stents, alternatives such as WRAPSODY (Merit Medical Systems, Inc) and Covera (Covera SG; Becton Dickinson) may be suitable, given their larger diameters and asymmetric flaring. A new device, Rapid Link (Terumo Aortic), combines a self-expandable stent with a surgical graft for suturing to the arch graft.^E4^ Although promising, it still requires manual sewing, limiting time savings. Its full potential lies in integration into hybrid FET prostheses without additional suturing, which will require broader size options. The DUETT Vascular Graft System represents another promising option for simplified supra-aortic anastomoses in FET. Its delivery through the arch-graft side-branch is currently under investigation (NCT06253143) and, if approved by the Food and Drug Administration, it may become a valuable on-label alternative.^E5^ The modified SAVSTEB technique presented here represents a step toward sutureless management of the supra-aortic vessels during hybrid aortic arch surgery.

## Conclusions

The modified SAVSTEB reduces systemic and cardiac arrest times, ensures good hemostasis, and could be extended to all supra-aortic vessels if appropriate stent sizes become available.

## Conflict of Interest Statement

The authors reported no conflicts of interest.

The *Journal* policy requires editors and reviewers to disclose conflicts of interest and to decline handling or reviewing manuscripts for which they may have a conflict of interest. The editors and reviewers of this article have no conflicts of interest.
